# Supplementary material for: Ranolazine Counteracts Strength Impairment and Oxidative Stress in Aged Sarcopenic Mice
Source: Metabolites. 2022 Jul 18;12(7):663. doi: 10.3390/metabo12070663 (PMC9316887; doi:10.3390/metabo12070663)
Supplement: Supplementary file 1 [file metabolites-12-00663-s001.zip › metabolites-1737842-supplementary.pdf]

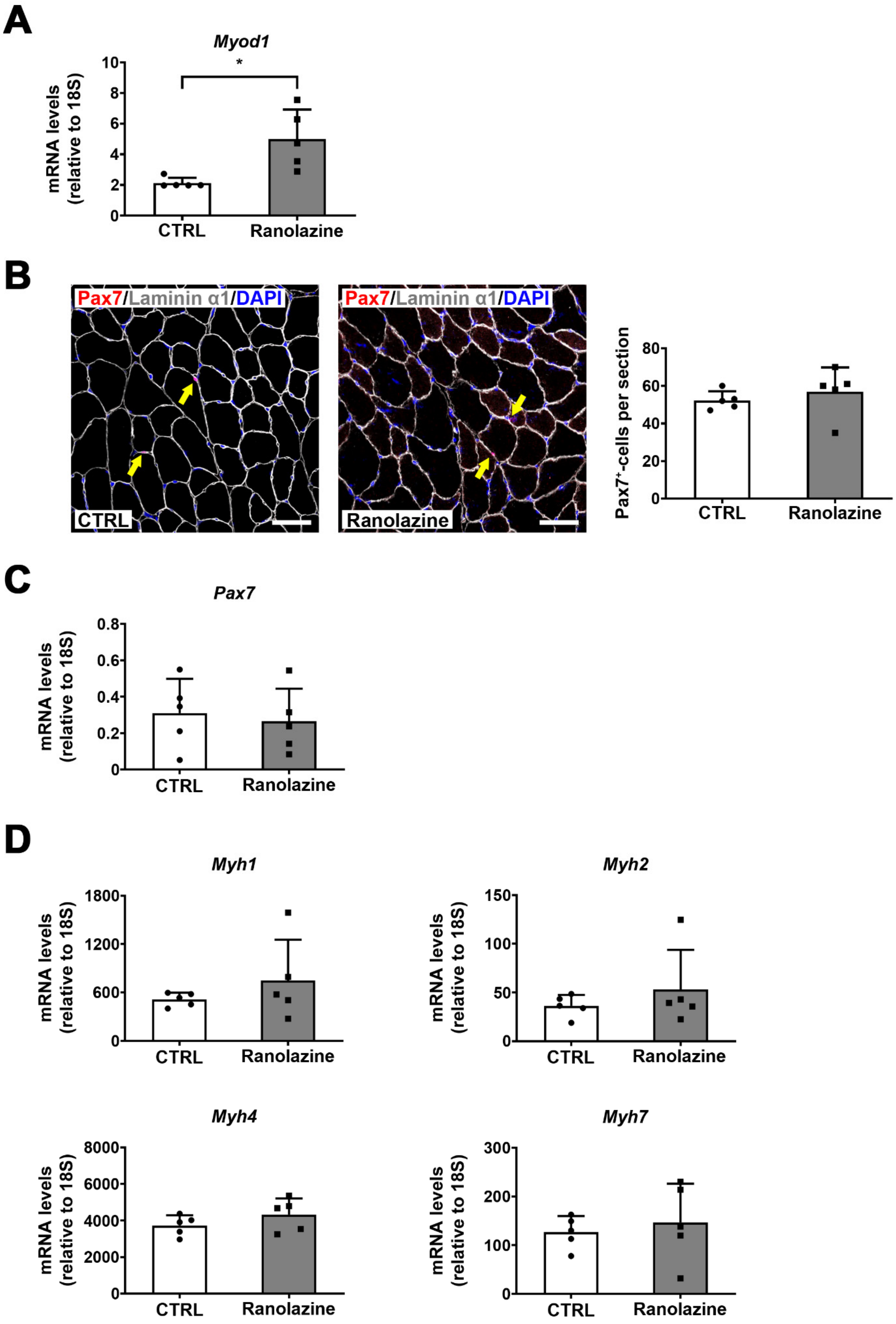

A
Myod1
*
mRNA levels (relative to 18S)
10
8
6
4
2
0
CTRL
Ranolazine
B
Pax7/Laminin α1/DAPI
CTRL
Ranolazine
Pax7⁺-cells per section
80
60
40
20
0
C
Pax7
0.8
0.6
0.4
0.2
D
Myh1
1800
1200
600
Myh2
150
100
50
Myh4
8000
6000
4000
2000
Myh7
300
200
100

FIGURE S1. Ranolazine up-regulates *Myod*1 expression in aged mice, but do not affect the expression of *Pax7* and *Myosins*. A) qPCR expression analysis of *Myod1* on whole TA muscles excised from 24 month-old mice treated or not treated with Ranolazine. Data are reported as relative to the housekeeping gene 18S. Values are means ± SD. N = 5 for both experimental conditions. Unpaired t-test was used for comparison. * = $p < 0.05$. **B)** Representative images of 8-μm-thick TA cryosections of old mice treated or not treated (CTRL) with Ranolazine stained with anti-Pax7 (red) and anti-Laminin α1 antibodies (grey). Nuclei were counterstained with DAPI (blue). Yellow arrows point at Pax7-positive cells (i.e. satellite cells). Scale bar = 50 μm. The histogram displayed the average of $Pax7^{+}$ cells per muscle section. **C)** qPCR expression analysis of *Pax7* on whole TA muscles excised from 24 month-old mice treated or not treated with Ranolazine. **D)** qPCR expression analysis of adult Myosins (*Myh1*, *Myh2*, *Myh4* and *Myh7*) on whole TA muscles excised from 24 month-old mice treated or not treated with Ranolazine. Data are reported as relative to the housekeeping gene 18S. Values are means ± SD. N = 5 for both experimental conditions. Unpaired t-test was used for comparison.
